# Evaluation of the phenotypic test and genetic analysis in the detection of glucose-6-phosphate dehydrogenase deficiency

**DOI:** 10.1186/1475-2875-12-289

**Published:** 2013-08-21

**Authors:** Duangdao Nantakomol, Rick Paul, Attakorn Palasuwan, Nicholas PJ Day, Nicholas J White, Mallika Imwong

**Affiliations:** 1 Department of Clinical Microscopy, Faculty of Allied Health Sciences, Chulalongkorn University, Bangkok, Thailand; 2 Institut Pasteur, Unit of Functional Genetics of Infectious Diseases, Paris, France; 3 Mahidol Oxford Research Unit, Faculty of Tropical Medicine, Mahidol University, Bangkok, Thailand; 4 Centre for Tropical Medicine, Churchill Hospital, Oxford, UK; 5 Department of Molecular Tropical Medicine and Genetics, Faculty of Tropical Medicine, Mahidol University, Bangkok, Thailand

**Keywords:** Glucose-6-phosphate dehydrogenase deficiency, Fluorescent spot test, Methaemoglobin reduction test, Enzymatic assay, Cytochemical method

## Abstract

**Background:**

Glucose-6-phosphate dehydrogenase (G6PD) deficiency is particularly prevalent in historically malaria-endemic countries. Although most individuals with G6PD deficiency are asymptomatic, deficiency can result in acute haemolytic anaemia after exposure to oxidative agents. A reliable test is necessary for diagnosing the deficiency to prevent an acute haemolytic crisis following, for example, anti-malarial treatment. The aim of this study was to investigate which method was the best predictor of this disorder.

**Methods:**

The present study investigated four G6PD activity detections (fluorescence spot (FS), methaemoglobin reduction (MR), biochemical and cytochemical test). These methods accompanied with mutation analysis of blood samples were taken from 295 apparently healthy individuals with unknown G6PD deficiency status.

**Results:**

Molecular characterization of 295 Thai adults revealed an overall prevalence of 14.2%. The G6PD Viangchan (871 G>A) was the most common (83.3%), followed by G6PD Mahidol (487G>A) (11.9%), and G6PD Union (1360 C>T) (4.8%). There were two cases of G6PD deficiency carrying the double mutations of Viangchan (871G > A)-Mahidol (487G > A) and Viangchan (871G > A)-Union (1360C > T). In comparison, the prevalence of G6PD deficiency was 6.1% by FS test and 7.1% by MR test. G6PD activity was 11 ± 2.5 IU/gHb in non-deficient females (mean ± SD), and 10.9 ± 0.6 IU/gHb in non-deficient males. The upper and lower limit cut-off points for partial and severe deficiency in adults were 5.7 IU/gHb (60% of the normal mean) and 0.95 IU/gHb (10% of the normal mean), respectively. All hemizygote, homozygote and double mutations were associated with severe enzyme deficiency (the residual enzyme activity <10% of the normal mean), whereas only 14.3% of the heterozygote mutations showed severe enzyme deficiency. Based on the cut-off value <5.7 IU/gHb, the quantitative G6PD assay diagnosed 83% of cases as G6PD-deficient. Using a cut-off number of negative cell >20% in the cytochemical assay to define G6PD deficiency, the prevalence of G6PD deficiency was closest to the molecular analysis (12.9% G6PD-deficient) compared to the others methods.

**Conclusion:**

The cytochemical method is a significant predictor of this disease, while FS and MR test are recommended for the detection of severe G6PD deficiency in developing countries.

## Background

Glucose-6-phosphate dehydrogenase (G6PD) is a housekeeping enzyme that catalyzes the first and rate-limiting step in the pentose phosphate pathway. Its key role in metabolism is to provide reducing power in the cytoplasm in the form of NADPH. This role is particularly important in red blood cells where NADPH serves as an electron donor for detoxification of hydrogen peroxide via reduced glutathione, and its production is crucial for the protection of cell from oxidative stress [1]. G6PD deficiency is the most common congenital enzyme deficiency in man, present in over 400 million people worldwide [2]. The human G6PD gene is located on Xq28, therefore full manifestation of the defective gene is observed in the male hemizygote and the female homozygote. In the female heterozygotes, a mixed population of normal and enzyme-deficient cells can be found [3]. Discrimination between hemizygously deficient males or homozygously deficient females and non-deficient individuals, on the basis of the determination of G6PDH activity in haemolysate, remains insufficient.

Expression of G6PD activity in heterozygote females is dependent on the degree of lyonization and of expression of the abnormal G6PD variant. Although most individuals with G6PD deficiency are asymptomatic, exposure to oxidant drugs, such as the anti-malarial drug primaquine, may induce haemolysis [4-6]. The broad geographical distribution of this defect and high prevalence in developing countries make it important for many counties to adapt tests that are simple and inexpensive. Rapidly ascertaining the G6PD status of a person is desirable when one is considering use of a drug contra-indicated in patients with G6PD deficiency. The semi-quantitative fluorescent spot test (FS test) and methaemoglobin reduction test (MR test) are widely used for population screening. These screening tests are considered to be antiquated methods of qualitative analysis of G6PD with low specificity and sensitivity [7,8] and as such, many are sceptical about the reliability of the outcome of the result of the methodology. Moreover, the semi-quantitative screening test is not reliable in detecting partially deficient individuals [9].

A sensitive cytochemical staining method has been developed for measuring G6PD activity in female heterozygous individuals [10]. Such a cytochemical assay offers a potentially good tool for discrimination among G6PD normal and heterozygously, hemizygously, and homozygously G6PD-deficient patients by the analysis of individual cells. Advances in molecular technology have allowed the molecular characterization of G6PD gene in any population to be carried out with relative ease [11]. However, there exist a large number of genetic mutations that have differing impact on G6PD activity. Previous studies have established the molecular abnormalities responsible for G6PD deficiency in several ethnic groups in Southeast Asia [12-17]. However, there has been no comparative study where all current G6PD diagnosis tests, especially phenotypic tests, were used to detect all cases of G6PD variants most commonly found in Thailand, including G6PD Viangchan (871G > A), Canton (1376G > T), Mahidol (487G > A), Kaiping (1388G > A), and G6PD Union (1360C > T) [16]. Therefore, the aim of this study was to compare the results of the semi-quantitative method, the enzymatic assay, the cytochemical method to a molecular method and identify which method was the best predictor of this disorder.

## Methods

### Sample collection

A total of 295 apparently healthy people with unknown G6PD deficiency status aged 18–50 years were recruited. There were 67 males and 228 females. All subjects had no concurrent infection and none had been hospitalized for at least three months. The research protocol was approved by the Ethical Review Committee for Research Involving Human Subjects in Research, Chulalongkorn University, in accordance with the International Conference on Harmonization-Good Clinical Practice (ICH-GCP). After obtaining informed consent, five milliliters of blood were obtained by venipuncture and placed into two tubes containing heparin and acid-citrate-dextrose (ACD), respectively. Blood samples were stored at 4°C until used. The FS test and MR test were performed on heparinized blood, whilst the G6PD activity assay (biochemical and cytochemical-based assay) was performed on ACD blood.

### DNA extraction and Identification of G6PD mutations

DNA was extracted from G6PD-deficient blood samples by using Qiaquick® Blood DNA extraction kit (Qiagen, Germany) according to manufacturer’s recommendations. In analysing the DNA, the strategy adopted was to screen for the five known G6PD variants that have been previously reported in Thai G6PD-deficient individuals: G6PD Mahidol 487, G>A; G6PD Viangchan 871, G>A; G6PD Kaiping 1388, G>A; G6PD Canton 1376, G>T; G6PD Union 1360, C>T. All were analysed with polymerase chain reaction (PCR)-restriction fragment length polymorphism (RFLP) as previously described [16].

The mutations were assessed in exons 6, 9, 11, 12, 13 for the identification of the less frequent mutations. Exons were amplified using primer pairs designed using Primer 3 software [18]. DNA yield was measured using a Nanodrop device. The PCR product was detected by electrophoresis using a 2% agarose gel and UV transluminator provided with the gel documentation system (Bio-Rad, USA). DNA sequencing was carried out initially in one direction in all exons, using either the forward or the reverse primer. Fluorescence-based cycle labelling of PCR products was performed using the BigDye Terminator v3.1 cycle sequencing kit (Applied Biosystems, USA). The labelled products were subjected to analysis using the ABI Prism 3100 Genetic Analyzer (Applied Biosystems, USA) in accordance with the manufacturer’s instructions. All mutations and polymorphisms were confirmed by both reverse and forward primers. Sequencing results were analysed using Sequence Scanner 1.0 [19].

### Methaemoglobin reduction test

The MR test was performed with a minor modification of Brewer *et al.*[20]. Heparinized blood samples were used only if they were not older than 24 hours. The working reagent was composed of 180 mM sodium nitrite and 280 mM dextrose solution dissolved in 0.4 mM of methylene blue chloride solution. Preparation of incubation tubes: positive control tube: 0.1 ml of the combined sodium nitrite-dextrose reagent was added without adding methylene blue; negative control tube: 1 ml of whole blood was mixed with 0.1 ml of distilled water; unknown sample tube: 0.1 ml of the combined sodium nitrite-dextrose reagent and 0.1 ml of methylene blue reagent were mixed with 1 ml of whole blood. These mixtures were mixed gently by inverting each tube and all tubes were incubated at 37°C for three hours. After incubation, 0.1 ml aliquot from each of the three tubes was transferred into preloaded 10 ml of distilled water in a glass tube. The colour of the unknown sample tube was compared visually to the colours of the positive and negative tube and replicates were tested for each sample. The unknown sample was considered not deficient if the colour was clear red, identical to that of the negative control, or deficient if the colour was dark grey or brown, identical to the positive control.

### Fluorescent spot test (FS test)

This technique is based on the visual evaluation of fluorescence reduced NADPH when activated by UV light. The method detects the fluorescence of NADPH, which is proportional to G6PD activity, under UV light long-wave (365 nm). The FS test was performed on the heparinized blood using a commercial kit (R&D Diagnosis, Holargos, Greece). Ten microlitres of blood was first incubated with 200 μl of the reagent mixture and spotted onto filter paper. Fluorescence intensity was measured at the beginning (zero time), 5, 10, and 20 min after incubation of blood with reagent mixture. Fluorescence intensity was classified into two groups: normal activity (bright fluorescence) and deficiency (no fluorescence).

### Biochemical G6PD activity

Blood samples were stored at 4°C and used only if they were not older than 24 hours. For the quantitative evaluation of G6PD activity, the G6PD kit from BIOLABO SA was used. The kit utilizes the chemical reaction and the NADPH produced is measured at 340 nm. The method involved an elution stage for the lysis of red cells and an assay stage that involved incubation with reagents containing substrate and cofactor NADP, followed by photometric measurement of the kinetic reaction at 340 nm. The assay was performed according to the manufacturer’s instruction and analysed by spectrophotometer within 1 hour. Briefly, 2 ml of ACD-blood were centrifuged to remove the buffy coat and washed three times with cold normal saline. These washed red blood cells (RBCs) were measured for haemoglobin concentration, and then 200 μl of the washed RBCs was lyzed by adding 2 ml of haemolyzing solution. Lyzed RBCs were centrifuged at 2,500 rpm for 20 min. The haemolysate was used within 1 hour. The rate of NADPH production was measured at 340 nm at 37°C for 10 min. The change of optical density per minute was calculated to determine the activity of the G6PD enzyme. In this study, the G6PD activity was expressed in international units per gram haemoglobin (IU/gHb) using the following calculation:

$$G6\mathrm{PDH}\;\left(\mathrm{IU}/\mathrm{gHb}\right)=\frac{\left(\Delta \mathrm{Abs}/\min\times 5000\right)\;}{\mathrm{Hb}\;\left(g/\mathrm{dl}\right)}$$

### Cytochemical assay

In this test, G6PD activity causes staining of individual RBCs by the reduction of water-soluble, colourless tetranitro blue tetrasolium, in its dark-coloured formazan by NADPH [10,21]. Dark-purple granules are present in RBC that contains G6PD activity, whereas G6PD-deficient RBC remains unstained. The number of both positive (RBC with dark-purple) and negative stained cells (RBC with little or no staining) were counted at least 1,000 RBCs. Two ml of heparinized blood were stored at 4°C and tested within one week of collection. Cellular G6PD activity was performed with a minor modification of the prior study by van Noorden [10,21]. Briefly, 1 ml of whole blood was added to 9 ml of 180 mM freshly prepared sodium nitrite dissolved in 0.9% NaCl and incubated for 5 min at room temperature. After incubation, the suspension was centrifuged for 15 min at 2,500 rpm, and then 75 μl of pack cells were added to 125 μl of a freshly prepared solution of NADP^+^ in phosphate buffer and incubated for 10 min at room temperature. After incubation, the suspension was centrifuged under the same conditions and 2 ml of a freshly prepared solution of 0.025% glutaraldehyde in phosphate buffer were added to the packed cells. The fixation was performed at room temperature under continuous rotation for 30 min. After incubation, the reaction was stopped by washing the fixed cells three times for 3 min with phosphate buffer. The cells were resuspended in the same buffer, then 100 μl of this cell suspension was added to 1 ml of the incubation medium and incubated for 90 min at 46°C in the dark, with constant rotation**.** Cells were washed three times with the same buffer and cells were resuspended in 1 ml phosphate buffer. Finally, cells were analysed for formazan presence by light microscopy. A person was considered as normal when <20% negative cells were found and as heterozygous-deficient when between 20 and 80% of cells were negative. When >80% negative cells were observed, males were considered as hemizygously deficient and females as homozygously deficient.

### Statistical analysis

The efficacy of the screening test was evaluated by determining the number of true positive, true negative, false positive, and false negative. To compare the accuracy of each of the screening tests in its ability to reflect the true G6PD status, as defined by molecular analysis, the following were calculated: sensitivity, [a/(a + c)]×100; specificity [d/(b + d)] ×100; negative predictive value [a/(a + b)] ×100 and positive predictive value [a/(a + b)] ×100, where a = true positives, b = false positives, c = false negatives, and d = true negatives. Statistical analyses were carried out using SPSS 11.0 statistical programs (SPSS Corporation, Chicago, IL, USA). P <0.05 was considered statistically significant.

## Results

### Identification of G6PD mutations

At least one G6PD mutation was identified in 42 of the 295 individuals (14.2% prevalence rate). G6PD Viangchan (871 G>A) was the most common and was detected in 36 of the 42 individuals (83.3%). G6PD Mahidol (487 G>A) was found in four individuals (11.9%) and G6PD Union (1360 C>T) was found in two cases (4.8%) (Table 1).

**Table 1:** G6PD status based on molecular analysis and G6PD enzyme level among G6PD variants (VC; Viangchan, VCMH; Viangchan-Mahidol, VCUN; Viangchan-Union, MH; Mahidol. UN; Union)

**Gender**	**Total**	**Deficient**	**Number of cases (**$\bar{x}$**± SD of G6PD enzyme activity (IU/gHb))**				
			**VC**	**VCMH**	**VCUN**	**MH**	**UN**
Male	67	6	5 (0.35 ± 0.02)	-	-	1 (0)	-
Female	228	36	29 (4.29 ± 3.61)	1 (0.4)	1 (0.83)	3 (3.23 ± 2.03)	2 (2.72 ± 1.10)

Among the 36 females, G6PD Viangchan was the dominant variant (80.5%), followed by G6PD Mahidol (8.3%), and G6PD Union (5.6%); there were two cases of double mutations, Viangchan-Mahidol and Viangchan-Union. There was one G6PD Viangchan homozygote. The others G6PD variants were heterozygotes. In the six male G6PD-deficient individuals, five cases were G6PD Viangchan (83.3%) and one case of G6PD Mahidol (16.7%).

### Assessment of phenotypic test for G6PD deficiency by biochemical method

**Figure 1:**
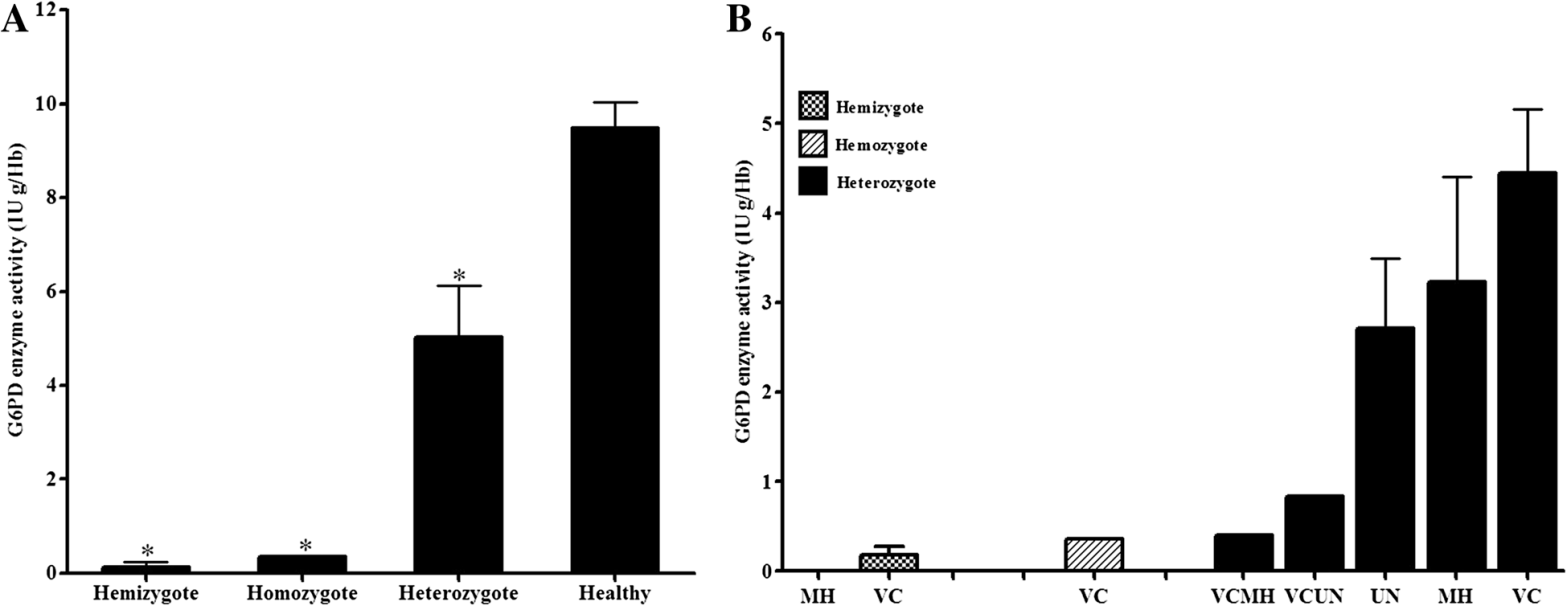
The means and standard errors of G6PD activity in (A) Red blood cells of G6PD normal, hemizygous, homozygous, and heterozygous G6PD-deficienct subjects (B) G6PD activity stratified by genotype; MH = Mahidol, VC = Viangchan, VCMH = Viangchan-Mahidol, VCUN = Viangchan-Union, UN = Union (all groups were significantly different *P **< 0.001).**

The biochemically determined G6PD activity in RBCs of G6PD normal and G6PD-deficient individuals are shown in Figure 1. In this study, the normal value of RBC G6PD activity was 11 ± 2.5 IU/gHb for G6PD normal females and 10.9 ± 0.6 IU/g Hb for G6PD normal males (Table 2). The reference range for normal G6PD activity in Thai adult female and male were 8.5-13.5 IU/gHb and 10.3-11.5 IU/gHb, respectively. Differences in mean normal G6PD activity between male and female Thai adults was not statistically significant (*P* = 0.49). In the hemizygous group, enzyme activity was very low ranging from 0 to 0.37 IU/gHb, whereas activity in the heterozygously G6PD-deficient group ranged between 0.4 and 12.13 IU/gHb. G6PD activity was significantly lower in the hemizygously (*P* < 0.001) and heterozygously deficient group (*P <* 0.001) as compared with the G6PD normal group. There was significant difference in the mean G6PD activity between heterozygously and hemizygously deficient groups (*P* < 0.001). Based on the cut-off value <5.7 IU/gHb, the quantitative G6PD assay diagnosed 83% (35 of 42) of G6PD-deficient individuals. Therefore, 17% of G6PD-deficient patients were misdiagnosed as normal.

**Table 2:** G6PD activity level in adult normal male and female

**Gender**	**Mean G6PD activity (IU/gHb)**	**Lower limit of severe deficiency (10% of mean value)**	**Upper limit of severe deficiency (60% of mean value)**
Male	10.9	1.09	6.54
Female	11	1.1	6.6
Total	9.5	0.95	5.7

### G6PD mutations and enzyme activity in different variants

In the female heterozygous group, the mean (SD) value of enzyme activity in G6PD Viangchan, G6PD Mahidol, and G6PD Union were 4.29 (3.61), 3.23 (2.03), and 2.72 (1.10) IU/gHb, respectively. The level of enzyme activity in G6PD-deficient individuals carrying the double mutations of Viangchan-Mahidol and Viangchan-Union were lower at 0.4 and 0.83 IU/gHb, respectively. The level of enzyme activity in homozygous G6PD Viangchan was 0.36 IU/gHb. Differences between groups were highly significant (*P* < 0.01). The mean enzyme concentration of males with the Viangchan variant was significantly lower at 0.35 ± 0.02 IU/gHb than female heterozygotes. The upper and lower limit cut-off points for partial deficiency in females were 6.6 IU/gHb (60% of the normal mean) and 1.1 IU/gHb (10% of the normal mean), respectively. The upper limit for severe deficiency in females was 1.1 IU/gHb. The upper and lower limit cut-off points for deficiency in males were 6.54 IU/gHb (60% of the normal mean) and 1.09 IU/gHb (10% of the normal mean), respectively [1,22]. The mean G6PD activity levels in normal, partial and severe G6PD-deficient groups are shown in Table 2. All hemizygotes, both with G6PD Viangchan and Mahidol had severe enzyme deficiency. Only 14.29% (five of 35) of the heterozygotes had as severe enzyme deficiency as the homozygote female.

### Assessment of phenotypic test for G6PD deficiency by FS and MR test

Among the apparently healthy 295 Thais adults, 21 individuals (7.1%) were found to be G6PD-deficient by MR test and 18 individuals (6.1%) by FS test. All of these subjects carried at least one of the identified G6PD variants. The sensitivity and specificity of both screening tests were assessed with respect to the genetic analysis (Table 3). The sensitivity of MR test to detect total G6PD deficiency was 0.5 and the specificity was 0.98. The sensitivity of FS test to detect total G6PD deficiency was 0.43 and the specificity was 1. All hemizygote, homozygote, and double mutant (100% or 9/9) G6PD-deficient individuals, whose G6PD activity was less than 0.83 IU/gHb, were correctly detected by both FS test and MR test. There were differences in the number of cases of G6PD Viangchan-deficient individuals detected by MR test and those with FS test. FS test detected G6PD deficiency in only 22% (8/36) of female adults with G6PD Viangchan mutation, while MR test detected G6PD deficiency in 42% (15/36). Among female heterozygotes diagnosed to have normal G6PD by the FS test, the enzyme level was in the range of 6.9 to 14 IU/gHb, greater than the upper limit (60% of the normal mean value).

**Table 3:** Assessing accuracy of the two screening tests in the identification of G6PD status

**Parameters**	**MR test**	**FS test**
True positive	21/42	14/42
True negative	249/253	253/253
False positive	4/253	0/253
False negative	21/42	28/42
Sensitivity	50%	43%
Specificity	98%	100%
Positive predictive value	84%	100%
Negative predictive value	92%	91%

### Assessment of phenotypic test for G6PD deficiency by cytochemical analysis

All hemizygotes, homozygotes and double mutants were correctly identified by the cytochemical method; the number of negative cells ranged from 95 to 98% (mean 96%). By comparison, the number of negative cells among female heterozygote and G6PD normal ranged from 18 to 67% (mean 54%) and 5 to 24% (mean 11%), respectively. The number of negative cells was significantly higher in all deficient groups as compared to G6PD normal (*P* <0.001). Additionally, the number of negative cells in the hemizygous group was significantly higher than the heterozygous group (*P* < 0.001).

When using the cytochemical criteria, individuals were considered as hemizygote and homozygote or severe deficient G6PD enzyme activity when the number of negative cells was >90%. When <15% negative cells were found, individuals were considered as normal and as heterozygous-deficient when between 20 and 80% of cells were negative. There was little overlap in the number of negative cells found in heterozygote individuals and G6PD normal (8.5% or 3/35). Using a cut-off number of negative cells <20% RBC to define overall G6PD deficiency status, the test was 91% sensitive and 87.5% specific. When the biochemical data were compared with the cytochemical data of all samples tested, a significant correlation was observed (r = 0.89; *P* <0.01). A significant correlation was also observed in samples of heterozygously deficient patients (r = 0.85; *P* <0.01). This correlation shows that biochemically determined G6PD activity correlates with the percentage of positive cells in heterozygote female subjects, who often have a G6PD activity within the normal range in the biochemical test.

## Discussion

G6PD deficiency is the most common, congenital, enzyme deficiency in humans, present in over 400 million people worldwide [2], and particularly in areas endemic for malaria. Routine screening for G6PD deficiency by semi-quantitative methods (FS test and MR test) is carried out in all hospitals in Thailand, but these do not diagnose G6PD deficiency in heterozygous women reliably. This study compared the results of semi-quantitative methods, an enzymatic assay and a cytochemical method to a molecular method to assess which method was the best predictor of this disease. Molecular characterization of 295 Thai adult subjects revealed that the G6PD Viangchan (871 G>A) was the most common (83.3%), followed by G6PD Mahidol (487 G>A) (11.9%), and G6PD Union (1360 C>T) (4.8%). There were two cases of G6PD deficiency carrying the double mutations between Viangchan (871G > A)-Mahidol (487G > A) and Viangchan (871G > A)-Union (1360C > T). This finding is in line with a previous study by Nuchprayoon *et al.* showing that the gene frequency of G6PD Viangchan is high among the Thais [16]. In contrast to a previous study, this study did not find G6PD Mahidol was the most common G6PD variant in Thailand [23]; this variant has recently been shown to be highly prevalent in the Karen group [24]. This contradictory result may be explained by the different population studied and the technique used in mutation analysis. The finding of this report is similar to that of Nuchprayoon *et al.*, who performed a genetic analysis [16], whereas the Panich *et al.* study used biochemical assays to study patients with acute haemolysis [23].

The overall (both male and female) prevalence of G6PD deficiency in this study was 7.1% using the semi-quantitative screening test, compared to 11.86% using the quantitative enzymatic assay, 12.9% using the cytochemical method, and 14.2% in the molecular analysis. The G6PD activity of 33.3% (14 of 42) of individuals carrying a mutation had <10% of the normal mean or severe enzyme deficiency. The remaining 28 cases had partial enzyme deficiency and all were misdiagnosed as normal by both the FS and MR tests. For any of the G6PD-variant groups, there was no hemizygous-deficient male case with partial enzyme deficiency; all G6PD-deficient males thus had severe enzyme deficiency. The FS and MR tests correctly diagnosed all male G6PD deficiency and the female severe deficiency individuals, who were homozygote double mutants. However, in the group of heterozygously G6PD-deficient females, the FS and MR test detected G6PD deficiency with nearly a 50% reduced success rate as compared to the molecular analysis. Thus the semi-quantitative screening test failed to diagnose 50% of G6PD deficiency and these cases were found to be exclusively females. It appears that although the semi-quantitative test can diagnose severe G6PD deficiency, it is not able to diagnose a substantial proportion of heterozygous G6PD-deficient individuals, who are nevertheless partially deficient, with G6PD activity ranging from 10 to 60% of normal.

Although genetic analysis provides a robust method of detecting known G6PD-variants, the technique is currently not sufficiently developed for routine screening; not only are there many variants to consider, there are likely several more that have yet to be identified. The cytochemical assay was a significant predictor of this disease and should be considered as a candidate to replace current methods. This study established the normal range, the mean and the standard deviation for G6PD activity for Thai adults. The overall means for G6PD-deficient and normal adults were 2.5 IU/gHb and 9.5 IU/gHb, respectively. It is crucial to determine the 60% cut-off point for mean normal residual G6PD activity to diagnose G6PD deficiency and determine the 10 to 60% range to establish partial deficiency. The partial deficiency ranges for G6PD activity in adult was 0.95-5.7 IU/gHb. The one case of partial deficiency, diagnosed as deficient by the semi-quantitative screening test, was a G6PD Viangchan heterozygote whose enzymatic level was 1.94 IU/gHb (20.4% of normal). It appears that the semi-quantitative screening test could only detect cases with the residual RBC enzyme activity lower than a cut-off point that is lower than 1.94 IU/gHb. The findings in this study are similar to those of Reclos *et al.* where 20% normal activity was set as the cut-off point for diagnosing G6PD deficiency [9]. Generally, G6PD deficiency is accepted when the activity is <1.5 IU/gHb [22]; this criterion may not be suitable for all populations, who may have different G6PD variants and hence levels of enzyme activity. The discrepancies on the cut-off value in quantitative assays should be defined in each population and tested.

G6PD deficiency is the most common inherited blood disorder and developing a standardized, robust methodology for population screening is vital for appropriate patient care. Most individuals with G6PD deficiency are asymptomatic but exposure to oxidant drugs, such as the anti-malarial drug primaquine, may induce haemolysis [4-6]. Primaquine is currently the only medication used for radical cure of *P. vivax* infection, which is frequently found in Thailand. Patients with G6PD deficiency have an increased susceptibility to hemolysis when given primaquine [25,26]. This potentially fatal clinical syndrome can be avoided if patients are tested for G6PD deficiency, adequately informed before being treated and placed under surveillance.

## Conclusion

The classical and perhaps best methods for detection of G6PD deficiency are the FS test and MR tests, which are inexpensive qualitative tests that are relatively rapid and easy to conduct. The biochemical assay is a quantitative method and is a significant predictor of this disease, while FS and MR test are recommended for the detection of severe G6PD deficiency in developing countries.

## Competing interests

The authors declare that they have no competing interests.

## Authors’ contributions

DN drafted the manuscript and collected the samples. AP performed data analysis and interpretation. RP, ND, NW, and MI were involved in providing the conception, design of the study and revised the manuscript critically for intellectual content and approved the final version of the manuscript. All authors read and approved the final manuscript.
